# Genome-Wide Identification and Characterization of TALE Superfamily Genes in Soybean (*Glycine max* L.)

**DOI:** 10.3390/ijms22084117

**Published:** 2021-04-16

**Authors:** Liang Wang, Xinyu Yang, Yingqi Gao, Shouping Yang

**Affiliations:** Soybean Research Institute, National Center for Soybean Improvement, Key Laboratory of Biology and Genetic Improvement of Soybean (General, Ministry of Agriculture), State Key Laboratory of Crop Genetics and Germplasm Enhancement, Jiangsu Collaborative Innovation Center for Modern Crop Production, College of Agriculture, Nanjing Agricultural University, Nanjing 210095, China; godspeedwangl@126.com (L.W.); xyyang11220311@126.com (X.Y.); gyq231121@163.com (Y.G.)

**Keywords:** soybean, TALE, genome-wide identification, evolutionary analyses, expression analyses, plant development, abiotic stresses

## Abstract

The three-amino-acid-loop-extension (TALE) superfamily genes broadly existed in plants, which played important roles in plant growth, development and abiotic stress responses. In this study, we identified 68 *Glycine max* TALE (GmTALE) superfamily members. Phylogenetic analysis divided the GmTALE superfamily into the BEL1-like (BLH/BELL homeodomain) and the KNOX (KNOTTED-like homeodomain) subfamilies. Moreover, the KNOX subfamily could be further categorized into three clades (KNOX Class I, KNOX Class II and KNOX Class III). The *GmTALE* genes showed similarities in the gene structures in the same subfamily or clade, whose coding proteins exhibited analogous motif and conserved domain compositions. Besides, synteny analyses and evolutionary constraint evaluations of the TALE members among soybean and different species provided more clues for GmTALE superfamily evolution. The *cis*-element analyses in gene promoter regions and relevant gene expression profiling revealed different regulating roles of *GmTALE* genes during soybean plant development, saline and dehydration stresses. Genome-wide characterization, evolution, and expression profile analyses of *GmTALE* genes can pave the way for future gene functional research and facilitate their roles for applications in genetic improvement on soybean in saline and dehydration stresses.

## 1. Introduction

The homeobox genes broadly exist in eukaryotes and play an important role in plant growth and development [1]. Early research divided the plant homeobox genes into 11 classes, including HD-ZIP, WOX, NDX, PHD, PLINC, LD, DDT, SAWADEE, PINTOX, KNOTTED-like homeodomain (KNOX) and BLH/BELL homeodomain (BEL1-like) [2]. Importantly, the KNOX and BEL1-like homeodomains can be further loop-connected by three extra amino acid residues and formed the three-amino-acid-loop-extension (TALE) superclass homeodomain, which is crucial for the regulations of diverse plant biological processes [3,4].

The KNOX proteins usually contain four domains: KNOX1, KNOX2, ELK, and KN homeodomain. In *Arabidopsis*, the *KNOX* genes were further classified into three classes (Class I, Class II and Class III) based on their gene structure characteristics and expression patterns [3,5,6,7]. There were four genes in the *Arabidopsis* KNOX Class I clade, *AtSTM*, *AtKNAT1*, *AtKNAT2* and *AtKNAT6*, which displayed distinct expression patterns and functions in the meristems [8,9,10]. For instance, *AtKNAT2* showed expression in the internal vegetative shoot apical meristem (SAM) and participated in carpel development [11]. Besides, *AtSTM* was proved to be associated with the shoot apical meristem formation during embryogenesis [12]. By contrast, the KNOX Class II genes were detected expressions in multiple tissues. Previously, the KNOX Class II genes, *AtKNAT7*, *PoptrKNAT7*, *GhKNAT7-A03* and *OsKNAT7* were reported to be crucial for cell elongation and secondary cell wall (SCW) biosynthesis in *Arabidopsis*, poplar, cotton and rice, respectively [13,14,15]. Interestingly, the KNOX Class III members were only found in dicotyledons. The KNOX Class III clade of *Arabidopsis* only contained one gene (*AtKNATM*) that affected the leaf polarity and leaf development [16]. Likewise, BEL1-like proteins also played important roles in plant growth and development. In *Arabidopsis*, there were 13 BEL1-like members and large-scale yeast two-hybrid experiments showed they generally were able to interact and form heterodimers with at least one KNOX protein [17,18]. An early study reported that the AtBLH1 protein cooperated with the AtKNAT3 protein to regulate *Arabidopsis* seed germination and seedling development [19]. Recently, some GhBEL1-like proteins were turned out to interact with the GhKNAT7 homologs and influenced the fiber SCW biosynthesis network in cotton [14]. In summary, the TALE superfamilies exhibited functional and regulating roles in plant development and different biological processes.

By comparison, gene function studies on soybean TALE superfamily members were rarely reported: GmBHL4 protein was demonstrated to heterodimerize with the GmSBH1 protein and modulated soybean plant growth, high temperature and humidity stress responses [20], and the ectopic expression of *GmKNT1* in *Arabidopsis* was able to alter of leaf morphology and flower identity [21]. Despite these studies, our understanding of TALE superfamily members in soybean is still very limited. With the rapid developments of the whole genome sequencing technologies, distinct soybean gene families like GRAS [22], HD-ZIP [23], WOX [24], WRKY [25], NAC [26], MYB [27] and ARF [28], were reported and systematically characterized. However, investigations on the *Glycine max* TALE (GmTALE) superfamily genes are still lacking. In this study, we identified 68 GmTALE superfamily members and explored their phylogenetic relations, gene structures, motif patterns, conserved domain patterns, chromosomal distributions and gene duplication events. Moreover, we conducted synteny analyses and evolutionary constraint evaluations of the TALE members among soybean and different species. Besides, the *cis*-elements in the promoter regions of the *GmTALE* genes were also investigated. Soybean is a worldwide economic crop that abundant in high-quality oil and protein [29,30], which is sensitive to the effects of abiotic stress and belongs to the group of crops that are less drought and saline tolerant [31]. Importantly, a recent study in poplar comprehensively analyzed the TALE gene family and turned out that 11 poplar *TALE* genes were responsive to saline stress [1]. In the current research, we carried out gene expression profiling in tissues or organs during soybean plant development, saline and dehydration stresses. To further explore the expression patterns of *GmTALE* genes in different tissues during saline and dehydration stresses, 12 representative genes from distinct GmTALE subfamilies or clades were selected and performed the quantitative RT-PCR analyses. In all, we conducted a comprehensive study on GmTALE superfamily members, which facilitated future functional studies on *GmTALE* genes and may directly or indirectly improve soybean genetic improvement in saline and dehydration stresses.

## 2. Results

### 2.1. Identification of TALE Superfamily Members in Soybean

In this study, 68 *GmTALE* genes were identified from the soybean *Wm82.a2.v1* genome on JGI Phytozome 13 (https://phytozome-next.jgi.doe.gov/, accessed on 15 April 2021), and referring to the nomenclature of *TALE* genes in poplar [1], we named them GmTALE1 to GmTALE68 according to their gene coordinate (Table S1). Among the identified *GmTALE* genes, 66 genes were located on the 20 distinct soybean chromosomes. The remaining two genes, *Glyma.U009200.2.Wm82.a2.v1* and *Glyma.U039200.1.Wm82.a2.v1*, were mapped on scaffold_21 and scaffold_44 of the soybean genome and were designated as *GmTALE67* and *GmTALE68*, respectively.

Concomitantly, the fundamental characteristics of the GmTALE family members were explored and listed in Table S1 including the open reading frame (ORF) length, the protein size (aa, amino acid), the isoelectric point (pI), the molecular weight (MW), the subcellular localization and the conserved domain compositions. As is shown in Table S1, the protein sizes of the GmTALE members varied from 83 aa (GmTALE61) to 795 aa (GmTALE43) and were corresponding to the MW ranged from 9514.28 Da to 87,161.19 Da. The pI values spanned from 4.43 (GmTALE28) to 8.94 (GmTALE44). Subcellular location predictions indicated that the GmTALE proteins (64 members) were mainly located in the nuclear region. Besides, three GmTALE members (GmTALE8, GmTALE20 and GmTALE29) were speculated in the cytoplasmic region, and one member (GmTALE61) was predicted in the extracellular region. And the gene coding sequences and protein sequences of the GmTALE members were listed in Table S2.

### 2.2. Phylogenetic Analysis and Classification of GmTALE Members

**Figure 1:**
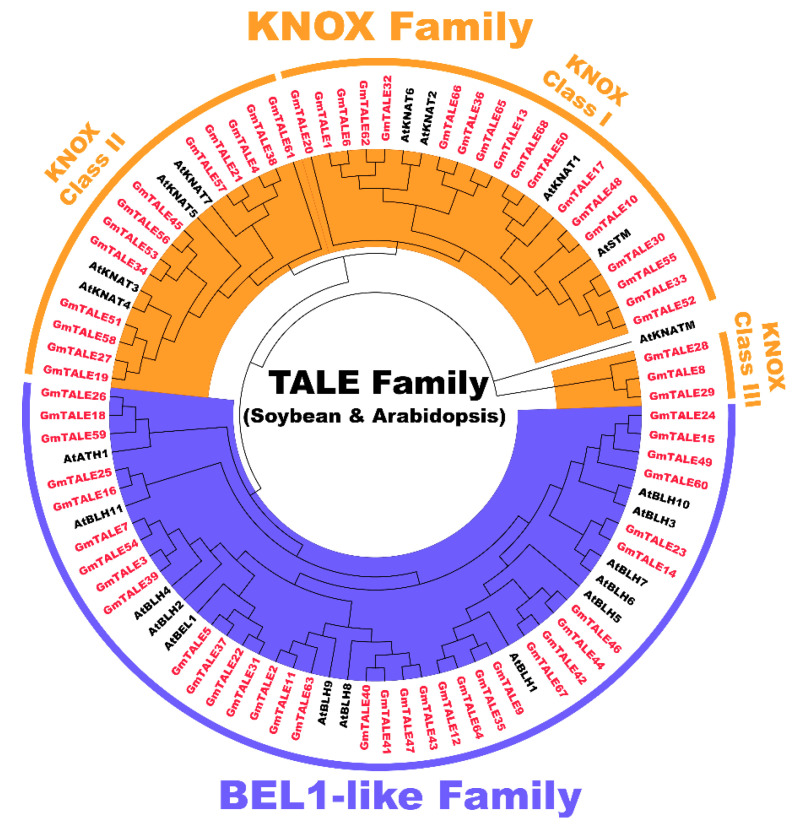
Unrooted phylogenetic tree of three-amino-acid-loop-extension (TALE) proteins in soybean and *Arabidopsis*. By using MEGA 7.0, the multiple protein sequences in two species were aligned with the MUSCLE method, and the tree was built used the maximum likelihood (ML) method with the best scoring JTT + G + I model. The tree was further categorized into the KNOX and BEL1-like subfamilies. All the GmTALE proteins have been emphasized in red.

To categorize the GmTALE proteins, the protein sequences of 68 identified GmTALE and the 22 reported AtTALE in *Arabidopsis* (Table S3) were aligned to build the maximum likelihood (ML) phylogenetic tree with the best scoring model JTT + G + I model (Figure 1). Referring to the classification in *Arabidopsis*, the 68 GmTALE family members were evenly divided into the BEL1-like subfamily and the KNOX subfamily (Table S1 and Figure 1) [4]. Besides, the 34 KNOX subfamily members were further classified into three classes, including KNOX-Class I (18 members), KNOX-Class II (13 members) and KNOX-Class III (three members) [4].

### 2.3. Gene Structures and Motif Compositions of GmTALE Members

**Figure 2:**
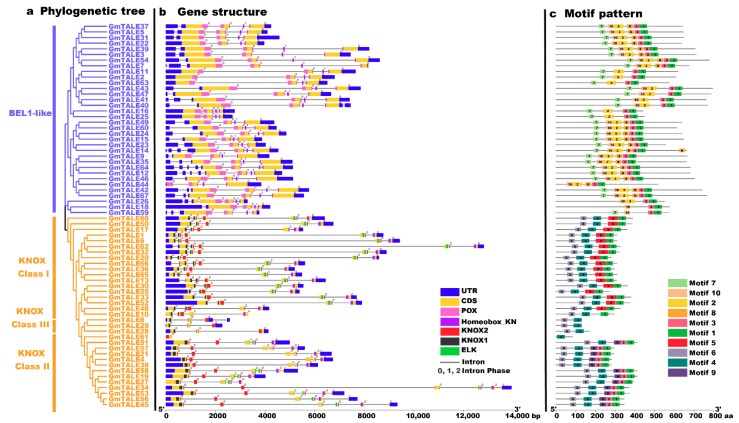
Phylogenetic relationships, gene structures and motif patterns of GmTALE members. (**a**) Phylogenetic clustering of GmTALE members. The phylogenetic tree was as built used the maximum likelihood (ML) method with the best scoring JTT + G model. (**b**) Gene structures of *GmTALE* genes. Blue boxes represent the untranslated 5′- and 3′-regions; yellow boxes indicate exons; black lines indicate introns. The numbers (0, 1, 2) indicate the phases of the introns. The diverse conserved domains were represented with different colored boxes. (**c**) The motif patterns GmTALE members. Besides, the length of relevant gene structures and motif components can be estimated with respective scales at the bottom of panels.

To gain more insight into the potential relationship between gene structure-function and the evolution process of GmTALE members, we investigated gene structures (the exon-intron patterns and the conserved domain compositions) of the identified GmTALE members (Figure 2). For the exon-intron patterns, the *GmTALE* genes displayed one to six exons (one *GmTALE* gene contains one exon; three *GmTALE* genes contain three exons, 39 *GmTALE* genes contain four exons, 17 *GmTALE* genes contain five exons and eight *GmTALE* genes contain six exons). *GmTALE* genes classified in the same subfamily showed similar gene structures (Figure 2b). Notably, most genes in the BEL1-like subfamily (except for *GmTALE26*) uniformly contained four exons. Moreover, the conserved domains of GmTALE members were also depicted (Figure 2b). As a whole, most GmTALE members (except for GmTALE61) contained the Homeobox_KN domains. While GmTALE members in the BEL1-like subfamily harbored POX domains, and the KNOX1, KNOX2 and ELK domains merely showed up in the KNOX subfamily. Importantly, the KNOX1 and KNOX2 domains were reported to compose the MEINOX domain, which mediated the formations of heterodimers between KNOX and BEL1-like proteins [14,32].

To better illustrate the conserved domain patterns of the *GmTALE* gene members, we carried out motif scanning by setting the motif amount as 10 with the MEME online software (https://meme-suite.org/meme/tools/meme, accessed on 15 April 2021). The detailed information of the ten MEME-motifs was listed in Table S4. And the Seq Logos of the MEME-motifs were depicted in Figure S1. A diagram was further constructed by the ten scanned MEME-motifs (named Motif 1–10) in Figure 2c. Importantly, GmTALE members within the same subfamily displayed similar motif compositions. Most BEL1-like subfamily members contained Motif 1, 2, 3, 7, 8, and 10, whereas the KNOX subfamily members were associated with Motif 1, 3, 4, 5, 6 and 9. In particular, the different clades of the KNOX subfamily exhibited diverse motif components. The KNOX Class I members contained Motif 1, 4, 5 and 6. By contrast, the GmTALE members in the KNOX Class II were associated with Motif 1, 3, 4, 6 and 9, while the KNOX Class III members were only coupled with Motif 4 and 6. Interestingly, distinct GmTALE members showed different motif constitutions, which paralleled to their conserved domain patterns. Taken together, we summarized the subordinations between conserved domains and the MEME-motifs: Motif 1, 3 and 8 corresponded to the Homeobox_KN domain; Motif 2, 7 and 10 were associated with the POX domain; Motif 5 and 9 were correlated to the ELK domain; Motif 6 was linked to the KNOX1 domain and Motif 4 was correlated to the KNOX2 domain (Table S4).

### 2.4. Chromosomal Distributions of GmTALE Genes

**Figure 3:**
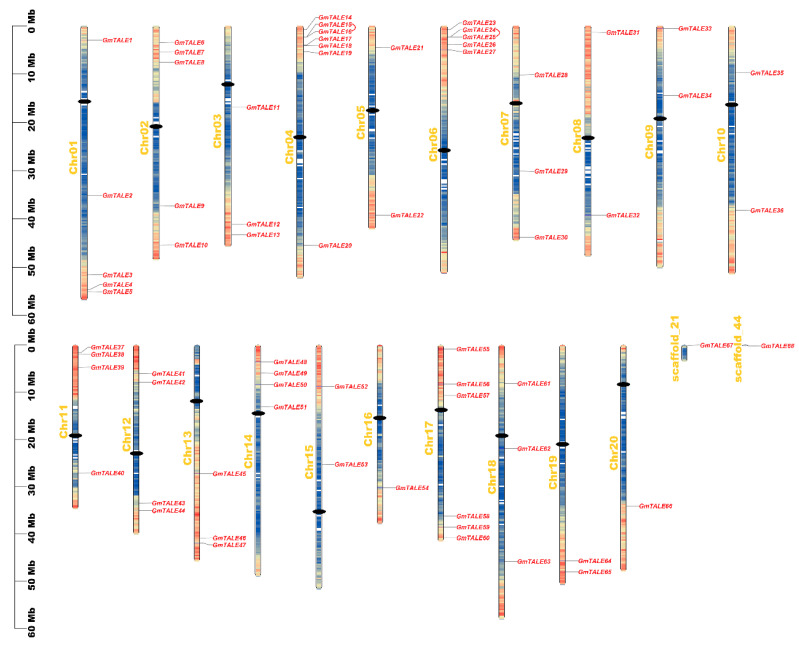
Chromosomal distributions of *GmTALE* genes. Chromosomal names were placed at the left. The scale on left is in megabases (Mb). Gradient colors from red to blue that attached to soybean chromosomes were corresponding from high to low gene density by setting the estimating hereditary interval as 300 kb. The red color represented high gene density, and the blue color represented low gene density. The blank regions on chromosomes were the genetic regions that lacked gene distributing information. And the centromeres were depicted with black dots. Besides, the tandemly duplicated *GmTALE* gene pairs were linked by red arcs.

As is illustrated in Figure 3, the *GmTALE* genes were unevenly distributed on the 20 chromosomes (Chr01 to Chr20), scaffold_21 and scaffold_44 of the soybean genome. Remarkably, Chr04 enclosed the most *GmTALE* genes (seven genes), whereas Chr20, scaffold_21 and scaffold_44 respectively contained one gene. And the chromosome or scaffold length did not present an apparent correlation with the number of *GmTALE* genes. To better revealing the distributing tendency of *GmTALE* genes, a series of gradient colors were endowed on soybean chromosomes or scaffolds, which were deduced from the gene numbers in the 300-kb genetic intervals on different soybean chromosomes or scaffolds (Table S5). Interestingly, most identified *GmTALE* genes tended to gather in the regions with high gene density.

### 2.5. Duplication, Syntenic and Evolutionary Analyses of GmTALE Genes

**Figure 4:**
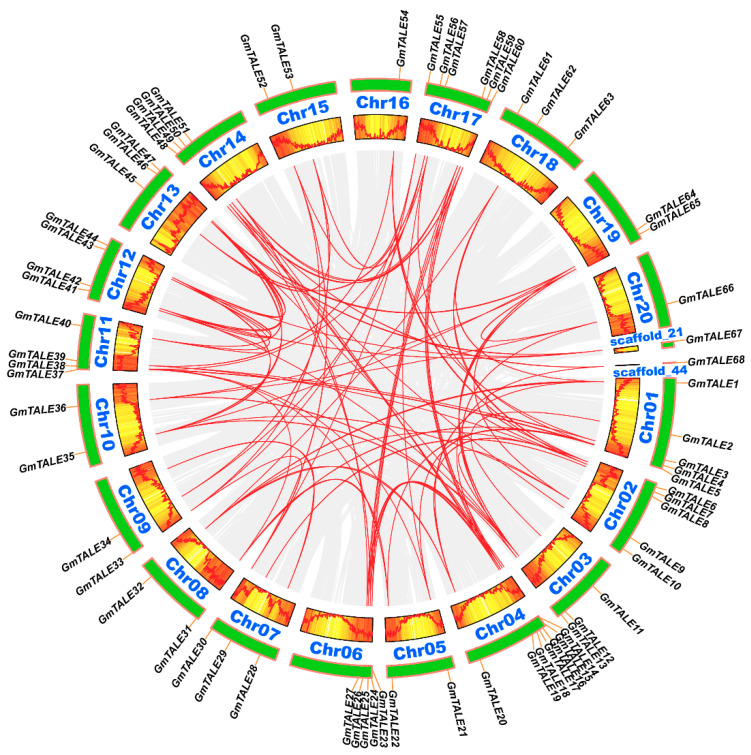
Circos plot displaying the collinearity of *GmTALE* homologous genes. Genome-wide collinear blocks were set as the background in gray and the duplicated *GmTALE* gene pairs were linked with red curves. Besides, each soybean chromosome was attached with 300-kb gene density information and depicted by heatmap and wave graph.

Tandem and segmental duplication events were regarded as two major driving forces in plant gene family expansions [33]. Referring to the former research, a 200-kb chromosomal region including two or more genes could be determined as a tandem duplication event [34,35]. In this study, two tandem duplication events associated with four *GmTALE* genes (*GmTALE15*/*GmTALE16* and *GmTALE24*/*GmTALE25*) were detected on Chr04 and Chr06 (Table S6). And the tandemly duplicated gene pairs were linked by the red arcs in Figure 3. By contrast, segmental duplications resulted in a large amount of duplicated chromosomal blocks in the genomes and often happened during polyploidization events with chromosome rearrangements [36]. In total 91 segmental duplication events associated with 63 *GmTALE* genes were dug throughout the soybean genome (Table S6). And the homologous *GmTALE* genes were further illustrated and jointed by red curves in the collinear Circos plot in Figure 4. Compared to the tandem duplications, the segmental duplication events mainly drove the expansion of GmTALE superfamily.

**Figure 5:**
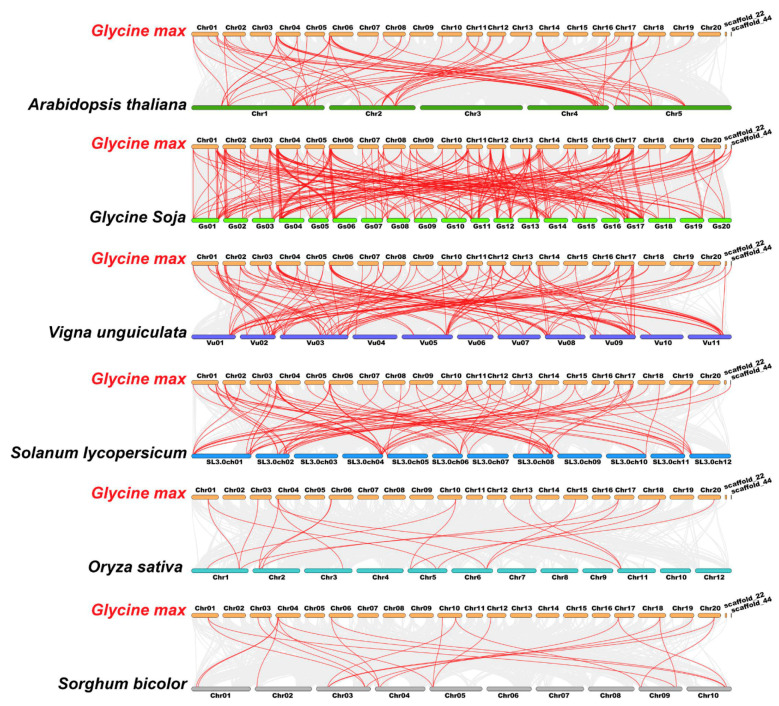
Syntenic relations of the TALE members among soybean and six representative plant species. Gray lines in the background indicated the collinear blocks within soybean and other plant genomes, and the red lines highlighted the syntenic *GmTALE* gene pairs.

To further explore the evolutionary clues of the TALE members among soybean and other species, four dicots (*Arabidopsis thaliana*, *Glycine soja*, *Vigna unguiculata* and *Solanum lycopersicum*) and two monocots (*Oryza sativa* and *Sorghum bicolor*) were applied for the synteny analyses (Table S7). Correspondingly, 233, 129, 84, 66, 22 and 19 *GmTALE* orthologous genes were identified in *Glycine soja*, *Vigna unguiculata*, *Solanum lycopersicum*, *Arabidopsis thaliana*, *Sorghum bicolor* and *Oryza sativa*, respectively. And the outputting results were integrated into the comparative syntenic schematics in Figure 5. Besides, we filtered the non-redundant *GmTALE* genes that exhibited the syntenic relationships among soybean and the other six species (Table S8). A total of 67 GmTALE members (except for GmTALE61) were found to be syntenic with *Glycine soja* (66 GsTALE members), *Vigna unguiculata* (32 VuTALE members), *Solanum lycopersicum* (23 SlTALE members), *Arabidopsis thaliana* (18 AtTALE members), *Sorghum bicolor* (10 SbTALE members) and *Oryza sativa* (nine OsTALE members). And an interactive Venn diagram of the non-redundant *GmTALE* genes throughout the different species was displayed in Figure S2. Notably, two GmTALE members (GmTALE2 and GmTALE12) had syntenic pairs throughout all the six species, which were further emphasized in bold in Table S8. The common orthologous gene pairs throughout distinct species may be useful for conducting relevant evolutionary studies of *GmTALE* genes.

**Figure 6:**
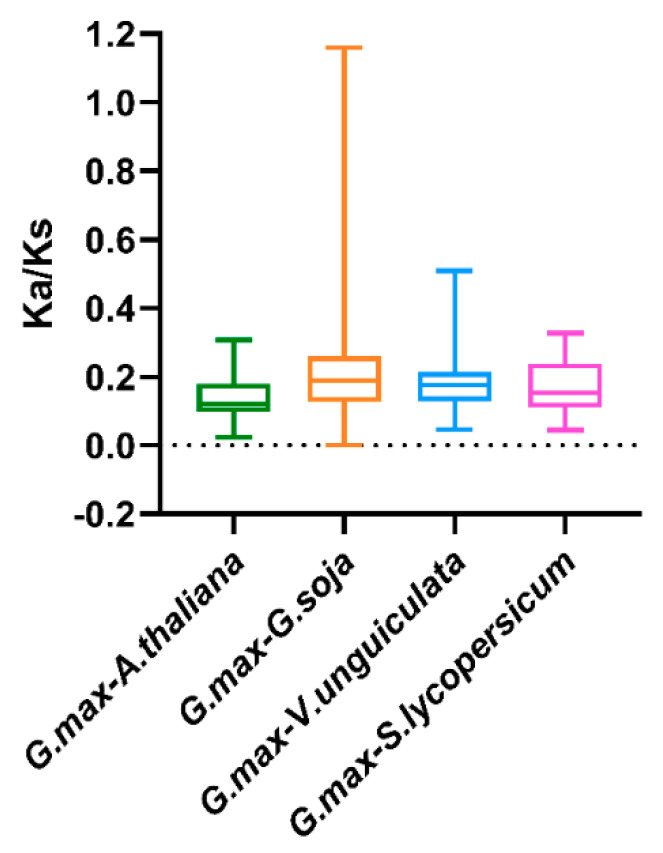
Box plot for the ratios of nonsynonymous to synonymous substitutions (Ka/Ks) in orthologous *TALE* gene pairs. The species names with the prefixes ‘*G. max*’, ‘*A. thaliana*’, ‘*G. soja*’, ‘*V. unguiculata*’ and ‘*S. lycopersicum*’ indicate *Glycine max*, *Arabidopsis thaliana*, *Glycine soja*, *Vigna unguiculate* and *Solanum lycopersicum*, respectively.

To illuminate the evolutionary constraints acting on the TALE superfamily, the Ka/Ks (non-synonymous substitution/synonymous substitution) ratios of the *TALE* orthologous gene pairs among soybean and the six species were calculated (Table S7). Moreover, the obtained Ka/Ks ratio values were depicted in the boxplot in Figure 6. Due to the lack of calculated Ka/Ks ratio values, the results among soybean and the two monocots were manually removed from the boxplot. As is shown in Figure 6, most of the orthologous *TALE* gene pairs displayed Ka/Ks < 1, indicating that the soybean TALE superfamily undergone strong purifying selective pressure during evolution among the dicots [22].

### 2.6. Cis-Element Analyses of Soybean GmTALE Genes

The *cis*-elements play essential roles in the transcriptional regulation of gene expression [37]. In this study, the promoter region sequences (the 2000 bp upstream sequences from gene initiation codons) of *GmTALE* genes were extracted for the *cis*-element analyses (Table S9). Moreover, the discovered *cis*-elements were proportionally displayed in Figure S3. Remarkably, the *cis*-elements like light responsive, auxin responsive, gibberellin responsive, abscisic acid responsive, MeJA responsive, defense and stress responsive, drought inducibility and anaerobic induction broadly existed in the gene promoter regions (Table S10). In summary, the acquired results demonstrated that *GmTALE* genes take potential roles in various biological processes, responses to plant hormones and abiotic stresses.

### 2.7. Expression Profiling of the GmTALE Genes in Different Soybean Tissues or Organs

**Figure 7:**
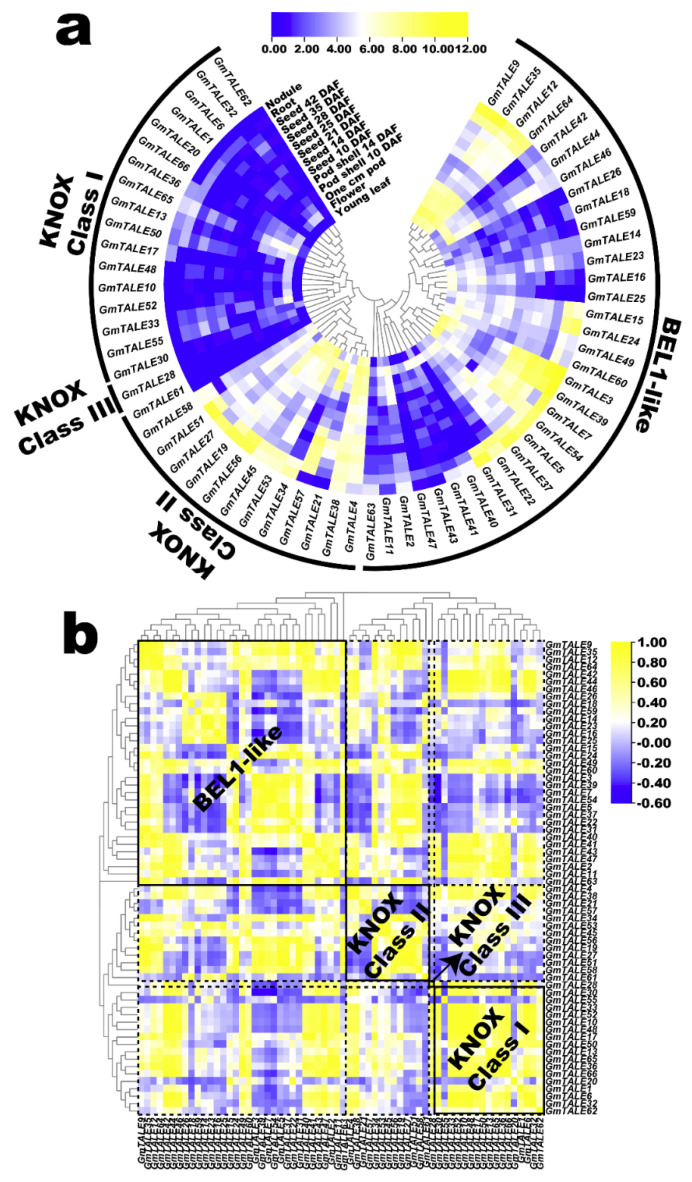
Expression profiling of *GmTALE* genes in various tissues or organs during soybean development. (**a**) Hierarchically clustered expression profiles of *GmTALE* genes in various tissues or organs during soybean development. DAF: days after flowering; cm: centimeter. (**b**) Gene expression correlation heatmap of the expressed *GmTALE* genes in various tissues or organs during soybean development. Yellow: positively correlated; blue: negatively correlated. The phylogenetic tree was as built used the maximum likelihood (ML) method with the best scoring JTT + G model.

The published RNA-seq data in SoyBase database (https://soybase.org/soyseq/, accessed on 15 April 2021) were adopted to explore the expression profiles of the *GmTALE* genes in different tissues or organs including young leaf, flower, one cm (centimeter) pod, pod shell 10 DAF (days after flowering), pod shell 14 DAF, seed 10 DAF, seed 14 DAF, seed 21 DAF, seed 25 DAF, seed 28 DAF, seed 35 DAF, seed 42 DAF, root and nodule [38]. The expression profiles of 64 *GmTALE* genes (except for *GmTALE8*, *GmTALE29*, *GmTALE67* and *GmTALE68*) were extracted (Table S11). To better illustrate the expression variations of the identified *GmTALE* genes, the acquired data were Log_2_ normalized to generate a heatmap in Figure 7a. In general, *GmTALE* genes in the distinct subfamilies presented diverse expression patterns. In the KNOX subfamily, most *GmTALE* genes in Class I and Class III universally showed low expression levels, whereas some KNOX Class II members (except for *GmTALE61*) tended to display relatively high gene expressions throughout tissues or organs. In the BEL1-like subfamily, the *GmTALE* genes in the same clade showed similar expression patterns and tissue-preferences. For example, *GmTALE3*, *GmTALE5*, *GmTALE7*, *GmTALE22*, *GmTALE31*, *GmTALE37*, *GmTALE39* and *GmTALE54* were clustered in the same clade and displayed relatively high expression levels in nodule and root. Another clade harbored *GmTALE18*, *GmTALE26* and *GmTALE59* universally displayed low expression levels (Figure 7a), whereas its adjacent clade consisted of four *GmTALE* genes (*GmTALE9*, *GmTALE12*, *GmTALE35* and *GmTALE64*) was found to be high expression levels by comparison.

**Figure 8:**
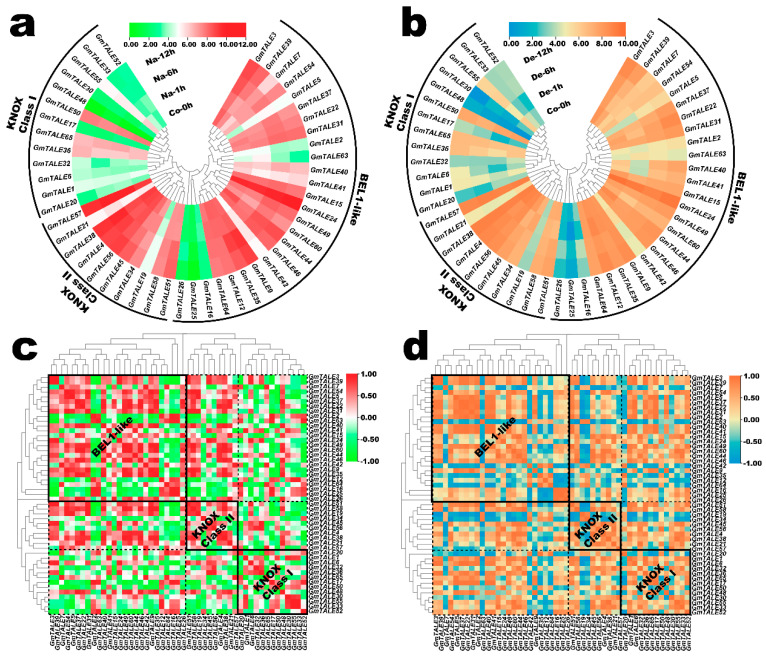
Expression profiling of *GmTALE* genes in the soybean root during saline stress and dehydration. (**a**) Hierarchically clustered expression profiles of *GmTALE* genes in the soybean root during saline stress. Na: saline stress; Co: control. (**b**) Hierarchically clustered expression profiles of *GmTALE* genes in the soybean root during dehydration. De: dehydration. (**c**) Gene expression correlation heatmap of the expressed *GmTALE* genes in the soybean root during saline stress. Red: positively correlated; green: negatively correlated. (**d**) Gene expression correlation heatmap of the expressed *GmTALE* genes in the soybean root during dehydration. Orange: positively correlated; blue: negatively correlated. The phylogenetic tree was as built used the maximum likelihood (ML) method with the best scoring JTT + G + I model.

Concomitantly, we calculated the correlation coefficients of the expressed *GmTALE* genes to further illustrate expression patterns in tissues or organs (Table S12). Then the acquired correlation coefficient matrix was recruited to draw a heatmap that clustered by the phylogeny of the GmTALE members (Figure 8b). To display the expression correlations among diverse clades, the correlation heatmap was divided into different blocks with the dotted lines based on the classification of the GmTALE subfamilies. Furthermore, we enclosed the BEL1-like and KNOX subfamilies with the solid boxes and labeled their names in bold in the heatmap. As a whole, both the positive and negative correlations broadly existed and interlaced among the GmTALE members of internal or external subfamilies. Notably, most *GmTALE* gene members in the KNOX Class I clade shared similar expression patterns and exhibited positive correlations. Comparably, eight *GmTALE* gene members (*GmTALE3*, *GmTALE5*, *GmTALE7*, *GmTALE22*, *GmTALE31*, *GmTALE37*, *GmTALE39* and *GmTALE54*) that clustered in one clade of the BEL1-like subfamily were broadly negatively correlated with the *GmTALE* gene members both internal and external subfamilies, which highlighted their different expression patterns. Overall, the disparity and similarity of gene expression patterns in distinct tissues or organs may manifest the potential regulating roles of the *GmTALE* genes during soybean plant development.

### 2.8. Expression Profiling of the GmTALE Genes in Soybean Root during Saline Stress and Dehydration

Previously, the *TALE* genes were reported to be associated with the saline stress response in poplar [1]. In the present study, we extracted the *GmTALE* gene expression profiles in the soybean root during saline stress and dehydration from the former published RNA-seq data [39]. As a result, the relevant expression profiles of 49 identified *GmTALE* genes were extracted including four-time points (0 h, 1 h, 6 h and 12 h) under the abiotic stress treatments (Table S13). Based on the Log_2_ normalized expression data, two heatmaps were respectively depicted corresponding to the *GmTALE* gene expressions during saline stress and dehydration in Figure 8a,b. In the KNOX Class I clade, most *GmTALE* genes (except for *GmTALE36*, *GmTALE50* and *GmTALE65*) displayed relatively low expression levels. By contrast, considerable gene members in the KNOX Class II clade (except for *GmTALE19*) and BEL1-like subfamily (except for *GmTALE2*, *GmTALE16*, *GmTALE25*, *GmTALE26* and *GmTALE63*) exhibited high gene expressions. Interestingly, the *GmTALE* genes with high or low transcript levels showed similarities during saline stress and dehydration. Moreover, the gene expression heatmap was simultaneously row-scaled with the zero-to-one method to show the expression variations of each *GmTALE* gene during saline stress and dehydration (Figure S4). As a whole, different *GmTALE* genes exhibited various expression patterns during the abiotic stresses.

Likewise, the gene expression correlation analyses during saline stress and dehydration were also conducted, and the acquired correlation coefficient matrixes were respectively displayed in Tables S14 and S15. Accordingly, the correlation heatmaps for *GmTALE* gene expression during saline stress and dehydration were built in Figure 8c,d. In general, positive and negative correlations of gene expression were universally existed and interlaced among the GmTALE members of internal or external subfamilies. However, the gene expression correlation patterns were diverse during the two abiotic stresses. For instance, during saline stress, in the BEL1-like subfamily, *GmTALE2*, *GmTALE16* and *GmTALE25* showed broadly negative correlations with most GmTALE members in the BEL1-like subfamily and the KNOX Class II clade compared to those during dehydration. In all, the *GmTALE* genes distinctly expressed and responded to saline stress and dehydration with different expression patterns.

### 2.9. Quantitative RT-PCR Investigations of GmTALE Gene Expression Patterns in Different Tissues during Saline Stress and Dehydration

**Figure 9:**
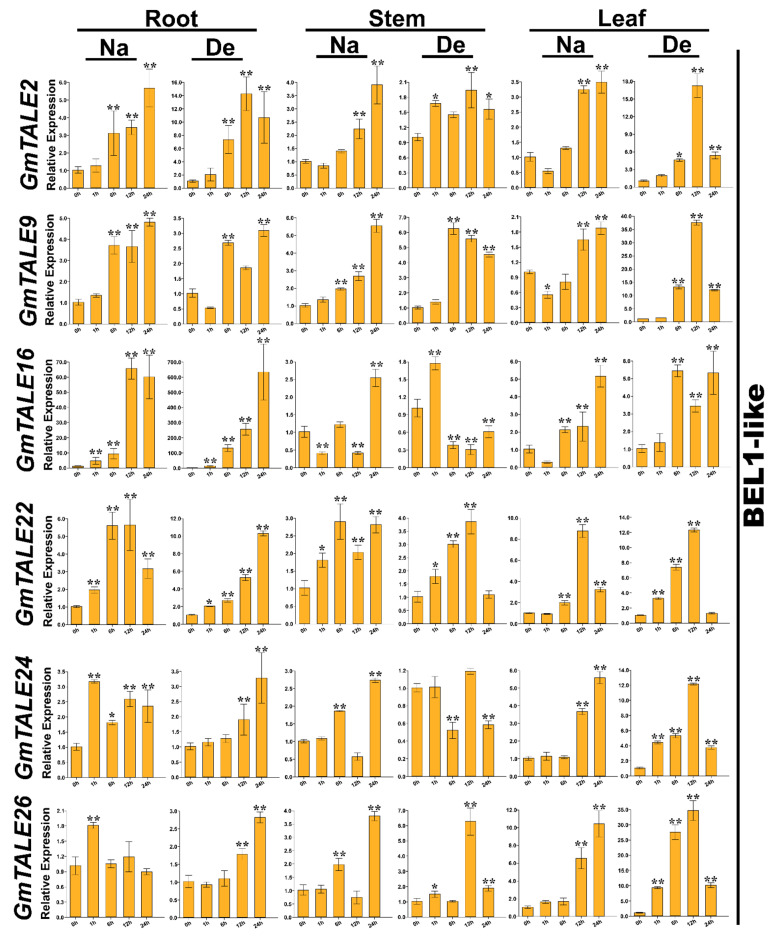
Quantitative RT-PCR analyses of the selected representative *GmTALE* genes in the BEL1-like subfamily during stress treatments throughout leaf, stem and root tissues. Data were normalized to the *GmActin* gene, and vertical bars indicated the standard deviations. The values represented the mean ± standard deviation (SD) of three independent replicates. Asterisks indicate the corresponding gene significantly up- or down-regulated compared with the 0-h statuses (* *p* < 0.05, ** *p* < 0.01, Student’s *t*-test). Na: saline stress; De: dehydration.

**Figure 10:**
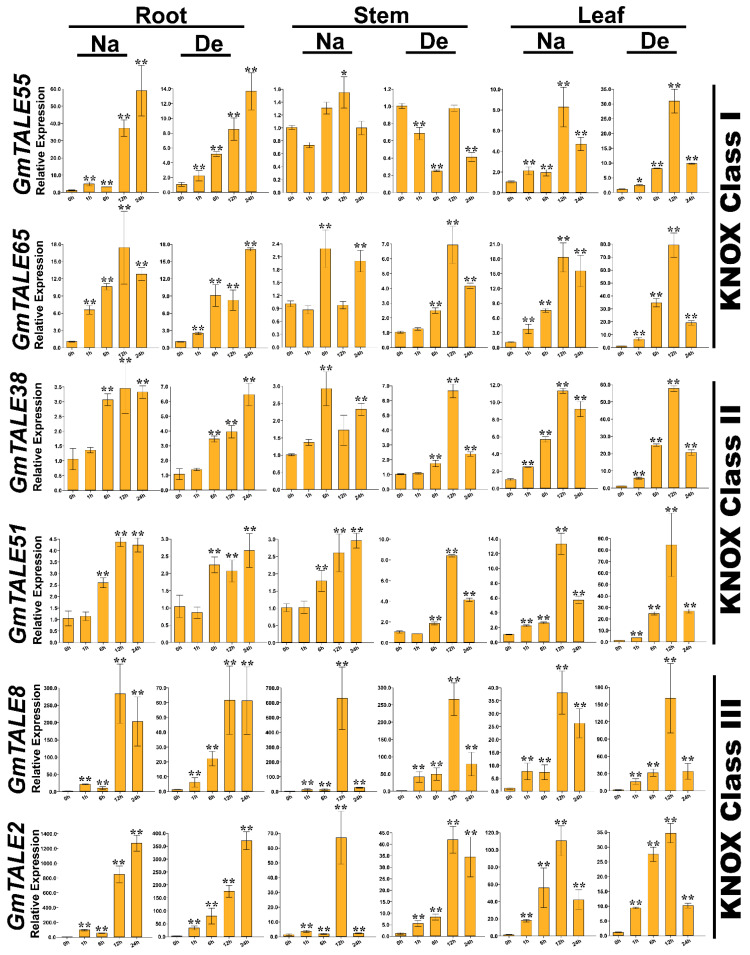
Quantitative RT-PCR analyses of the selected representative *GmTALE* genes in the KNOX subfamily during stress treatments throughout leaf, stem and root tissues. Data were normalized to the *GmActin* gene, and vertical bars indicated the standard deviations. The values represented the mean ± standard deviation (SD) of three independent replicates. Asterisks indicate the corresponding gene significantly up- or down-regulated compared with the 0-h statuses (* *p* < 0.05, ** *p* < 0.01, Student’s *t*-test). Na: saline stress; De: dehydration.

Taking the gene expression patterns during saline stress and dehydration (Figure S2) as well as the *cis*-elements in gene promoter regions (Figure S3) into consideration, 12 representative *GmTALE* genes, whose expression levels were relatively high at diverse time points, were carefully selected from different GmTALE subfamilies or clades and carried the quantitative RT-PCR analyses. The specific primers of the selected genes were designed and listed in Table S16. Quantitative RT-PCR assays were conducted to investigate the selected gene expression patterns in leaf, stem and root tissues during saline stress and dehydration (Figure 9 and Figure 10).

**Figure 11:**
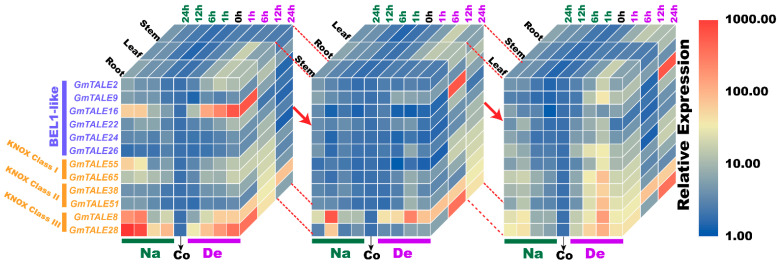
Cubic heatmaps for representative selected *GmTALE* genes during abiotic stress treatments throughout leaf, stem and root tissues. The quantitative RT-PCR results were integrated to depict the cubic heatmaps, which were emphasized and displayed by different tissue layers. Na: saline stress; De: dehydration; Co: control.

Compared to the transcriptome data, we additionally analyzed the selected *GmTALE* gene expressions in the leaf and stem tissues as well as explored the gene transcript levels after 24 h (24-h) stress treatments. Overall, the relative expressions of selected *GmTALE* genes significantly up-regulated at different time points, which indicated they were responsive to the saline and dehydration stresses but under distinct response patterns (Figure 9 and Figure 10). To better illustrate the diverse gene expression patterns of the selected *GmTALE* genes throughout tissues and different abiotic stresses, we integrated the obtained quantitative RT-PCR results and constructed a gene expression cubic heatmap in Figure 11. It is worth noting that the gene up-regulated levels under the abiotic stresses in stem tissue universally lower than those in root and leaf tissues (Figure 11). To sum up, the selected genes displayed distinct expression patterns during saline stress and dehydration in different soybean tissues, which may manifest their possible roles in responding to different abiotic stresses.

## 3. Discussion

The TALE superfamily genes were ubiquitously found in plant genomes, which were crucial for regulating plant development, growth and stress responses [1,14]. With the rapid developments of biotechnology and bioinformatic techniques in recent years, the TALE gene members in *Arabidopsis*, potato, poplar and cotton have been genome-wide identified and studied [1,4,14,40]. However, the genome-wide identification and characterization of soybean TALE superfamily members are still lacking. In this study, we carried out systematical identification and investigation on the GmTALE superfamily members, including their phylogenetic relationships, gene structures, conserved domains and motif patterns, gene chromosomal locations, gene duplication analysis, syntenic analyses, evolutionary constraints evaluations and *cis*-elements analyses in gene promoter regions. Besides, we also explored the expression patterns of the *GmTALE* genes in various tissues during soybean development, saline stress and dehydration.

Comparably, the numbers of TALE superfamily members were varied in species. Here, we obtained more *TALE* gene members in soybean (68 members) than those in *Arabidopsis* (22 members), poplar (35 members), *G. arboretum* (46 members) and *G. raimondii* (48 members) [1,4,14]. Whereas, compared to *G. barbadense* (88 members) and *G. hirsutum* (94 members), soybean had fewer *TALE* genes [14]. Polyploidy occurred in the majority of angiosperms and according to the overview of the sequenced plant polyploid genomes [41], we speculated that the numbers of *TALE* genes were associated both with the species genome sizes as well as their ploidy levels.

To explore the evolutionary correlations and classification of the 68 identified GmTALE proteins, we constructed the ML-phylogenetic tree with the best scoring model. Referring to the classification of TALE superfamily in *Arabidopsis*, the GmTALE members were equally divided into the BEL1-like subfamily and the KNOX subfamily, and the KNOX subfamily was further distributed into three classes. The TALE members in the same clade of different species may indicate their analogous biological functions [22]. Notably, each GmTALE subfamily or hereditary class had specific domains or motif combinations (Figure 2), which also support our classification results. Specific domains or motifs were reported to play important roles in DNA binding and protein interactions [42]. For instance, the POX domains were exclusively existed in plant proteins and associated with homeobox domains. In *Arabidopsis*, proteins containing the POX domains were included in the BEL1-like proteins and reported to interact with KNAT2 and KNAT5 proteins and affected plant development [4,17]. The ELK domain usually spanned about 21 amino acids and was dubbed for a highly conserved series of Glu, Leu, and Lys amino acids. It could function as a nuclear localization signal, which was also considered to act as a protein-protein interaction domain [32,43,44,45]. The KNOX1 and KNOX2 domains together formed the MEINOX region. Importantly, KNOX1 played a role in suppressing target gene expression. KNOX2, essential for function, was thought to be necessary for homo-dimerization [32]. The specific domain or motif patterns of GmTALE members may suggest they have different functions, which need to recruit future validations.

Similar to the cases in poplar and cotton, the protein sizes and molecular weights of the TALE members in the BEL1-like subfamily of soybean are much larger than those in the KNOX family (Table S1) [1,14]. Gene structure analysis showed that all *GmTALE* genes contain introns. Previous studies demonstrated that the introns are crucial for the evolution and generating of new gene family members [46,47]. Remarkably, considerable introns were located in the 5′ UTR (untranslated region) of BEL1-like subfamily genes compared to those of the KNOX subfamily (Figure 2). The intron distribution patterns of *GmTALE* genes may provide valuable clues for investigating the evolution of *GmTALE* genes. Moreover, *GmTALE* gene structures shared similarities in the same subfamily or clade, whereas, were distinct in different subfamilies. The characteristic divergencies and consistencies of the GmTALE members may also manifest their functional comparability and difference.

Plant genome evolutions often along with segmental and tandem duplications that resulted in the expansions of different gene families [48]. Gene duplication analysis turned out that most *GmTALE* genes were originated from segmental duplications (Table S6) that highlighted the important roles of segmental duplications in gene family expansions [33,49]. To further investigate the evolution clues for GmTALE members, four dicotyledons and two monocotyledons were recruited and performed the synteny analyses (Figure 5). We detected more *GmTALE* orthologous genes in dicotyledons than those in monocotyledons. Besides, *Glycine max* (soybean) and *Glycine soja* displayed the best synteny. The results indicated that the syntenies among TALE superfamily members may parallel to the evolutionary divergence of species. Importantly, GmTALE2 and GmTALE12 had syntenic pairs throughout dicotyledons and monocotyledons, which demonstrated that these orthologous pairs are conserved and may already exist before the ancestral divergence [22,35]. Moreover, the syntenic gene pairs among diverse species may be beneficial for evolutional research on GmTALE superfamily.

The *cis*-element analyses in gene promoter regions revealed the different roles of the identified *GmTALE* genes in regulating soybean development and responses to various abiotic stresses (Figure S3). By exploring the expression patterns of *GmTALE* genes during developments and abiotic stresses in various organs or tissues, we acquired a series of diverse transcript abundance (Figure 7a and Figure 8a,b) [38,39]. Importantly, considerable *GmTALE* genes in the BEL1-like subfamily and the KNOX Class II clade displayed high gene expressions, which may manifest they were possibly key genes in regulating these processes. Moreover, the gene expression correlations were also assessed and visualized (Figure 7b and Figure 8c,d). Correspondingly, the positive and negative correlations were broadly detected and varied in distinct *GmTALE* gene subfamilies. Interestingly, despite relevant *GmTALE* genes presented similarities in expressions (high or low) during saline stress and dehydration, whereas the gene expression correlation patterns of *GmTALE* genes during the stress treatments were different (Figure 8). Hence, we inferred that functional *GmTALE* genes may broadly play roles in responding to saline stress and dehydration while under different mechanisms.

Abiotic stresses, such as salinity, drought, high temperature, can decrease productivity and cause considerable losses in crop yields [50]. In poplar and soybean, relevant *TALE* genes were reported to be associated with the responses to distinct abiotic stresses [1,20,51]. In the current investigation, we further carried out the quantitative RT-PCR analyses to assess the responses of representative *GmTALE* genes to saline stress and dehydration in leaf, stem and root tissues (Figure 9, Figure 10 and Figure 11). Importantly, the cubic heatmap integrated a global view of the expression patterns of representative *GmTALE* genes responding to different abiotic stresses. It is worth noting that *GmTALE8* and *GmTALE28* up-regulated both during saline stress and dehydration throughout different tissues (Figure 11), which may indicate they were the key responsive genes to the two abiotic stresses. In general, the up-regulated levels of the selected genes in root tissue at distinct time points of the quantitative RT-PCR assays (Figure 9, Figure 10 and Figure 11) were higher than those of the former transcriptome data (Table S13 and Figure 8) [39]. Besides, the selected *GmTALE* genes universally presented higher expressions in the root and leaf tissues than in stem tissue during the stress treatments. Compared to the stem tissue, the root and leaf tissues may more sensitive to abiotic stresses. And the *GmTALE* genes might be extensively mobilized in root and leaf tissues to respond to the abiotic stresses. Taken together, the *GmTALE* genes have underlying regulatory roles in responding to abiotic stresses. And the variations in temporal and spatial expression patterns of the core functional *GmTALE* genes may be regarded as an effective regulation strategy in response to abiotic stresses.

This study provided a comprehensive investigation of GmTALE superfamily, which may be beneficial to gain insights into their biological functions. However, the current study only provided a preliminary characterization of *GmTALE* genes, and further functional validation should be carried out to understand the different roles of *GmTALE* genes in various biological processes.

## 4. Materials and Methods

### 4.1. Identification of Soybean TALE Superfamily Members

To identify the TALE superfamily members in soybean, we downloaded the soybean genome (the *Glycine max Wm82.a2.v1* version) and its annotation file from JGI Phytozome 13 with the accession number of ACUP02000000 (https://phytozome-next.jgi.doe.gov/info/Gmax_Wm82_a2_v1, accessed on 15 April 2021). The AtTALE protein sequences in *Arabidopsis* were obtained from the TAIR (https://www.arabidopsis.org/, accessed on 15 April 2021) and as the query sequences to extract the most representative GmTALE protein (the longest protein) sequences by TBtools software (https://github.com/CJ-Chen/TBtools/releases, accessed on 15 April 2021) [52]. The acquired GmTALE protein sequences were further verified by NCBI BLASTp (https://blast.ncbi.nlm.nih.gov/Blast.cgi?PROGRAM=blastp&PAGE_TYPE=BlastSearch&LINK_LOC=blasthome, accessed on 15 April 2021). The conserved domains of GmTALE proteins were explored in the NCBI-Conserved Domain database (https://www.ncbi.nlm.nih.gov/Structure/cdd/wrpsb.cgi, accessed on 15 April 2021). Proteins that lack TALE-associated domains were manually removed. The molecular weight (MW), isoelectric point (pI) and amino acid (aa) numbers of the identified GmTALE proteins were obtained from ExPASy (http://expasy.org/tools/, accessed on 15 April 2021) online tools. Besides, the CELLO software (http://cello.life.nctu.edu.tw/, accessed on 15 April 2021) was adopted for the *GmTALE* gene subcellular localization predictions.

### 4.2. Phylogenetic Analysis and Classification of GmTALE Members

The GmTALE and AtTALE protein sequences were together aligned by the MUSCLE method in MEGA 7.0 (https://www.megasoftware.net/, accessed on 15 April 2021) with the default parameters [53,54]. The aligned sequences were applied to build the phylogenetic tree that used the maximum likelihood (ML) method with the best scoring JTT + G + I model followed by the parameters: partial deletion, and 1000 bootstrap replications. Then the GmTALE proteins were classified referring to the category of TALE proteins in *Arabidopsis* [4]. By using FigTree v1.4.3 (http://tree.bio.ed.ac.uk/software/figtree/, accessed on 15 April 2021), the preliminary phylogenetic tree was visually examined, and different subfamilies were highlighted in distinct colors. Adobe Illustrator CC 2019 (https://www.adobe.com/products/illustrator.html, accessed on 15 April 2021) was adopted to modified the text sizes and draw different colored arcs surrounding different GmTALE subfamilies.

### 4.3. Gene Structure and Conserved Motif Analyses

Based on the soybean genome annotation file and acquired conserved domain information, we depicted the GmTALE gene structures by TBtools [52]. The MEME v5.1.1 online tool (http://meme-suite.org/tools/meme, accessed on 15 April 2021) was recruited for conserved motifs scanning by setting motif numbers as ten. Moreover, the Seq Logos of ten obtained MEME-motifs were visualized by TBtools. The output graphs were further modified by Adobe Illustrator CC 2019.

### 4.4. Chromosomal Location, Duplication and Synteny Analyses of GmTALE Genes

According to the soybean genome annotation file, we obtained the 300-kb hereditary interval gene densities and further transformed them into the gradient-colored heatmap on soybean chromosomes or scaffolds. The chromosomal locations of *GmTALE* genes were displayed by TBtools [52]. The segmentally and tandemly duplicated homologous *GmTALE* genes were detected by TBtools. In the gene chromosomal location plot and the collinear Circos plot, the duplicated *GmTALE* gene pairs were linked by the red arcs and cruves. To investigate the synteny among the TALE members of soybean and other species, we further downloaded the genome data and the gene annotation files of species including *Arabidopsis thaliana* (TAIR annotation release 10), *Glycine soja* (V1.1), *Vigna unguiculata* (V1.1), *Solanum lycopersicum* (ITAG3.2), *Oryza sativa* (MSU annotation release 7.0) and *Sorghum bicolor* (V3.1.1). The genome and its annotation file of *Arabidopsis thaliana* were downloaded from JGI Phytozome 13 (https://phytozome-next.jgi.doe.gov/info/Athaliana_TAIR10, accessed on 15 April 2021). The genome and its annotation file of *Glycine soja* were downloaded from JGI Phytozome 13 (https://phytozome-next.jgi.doe.gov/info/Gsoja_v1_1, accessed on 15 April 2021). The genome and its annotation file of *Vigna unguiculata* were downloaded from JGI Phytozome 13 (https://phytozome-next.jgi.doe.gov/info/Vunguiculata_v1_1, accessed on 15 April 2021). The genome and its annotation file of *Solanum lycopersicum* were downloaded from JGI Phytozome 13 (https://phytozome-next.jgi.doe.gov/info/Slycopersicum_ITAG3_2, accessed on 15 April 2021). The genome and its annotation file of *Oryza sativa* were downloaded from JGI Phytozome 13 (https://phytozome-next.jgi.doe.gov/info/Osativa_v7_0, accessed on 15 April 2021). The genome and its annotation file of *Sorghum bicolor* were downloaded from JGI Phytozome 13 (https://phytozome-next.jgi.doe.gov/info/Sbicolor_v3_1_1, accessed on 15 April 2021). The synteny analyses and the depiction of syntenic graphs of multiple species were performed by TBtools. Furthermore, the overviews of the *GmTALE* genes that contained orthologous genes in different species were presented by the Venn diagram. The ratios of nonsynonymous substitution (Ka) to synonymous substitution (Ks) of *GmTALE* orthologous gene pairs were calculated by TBtools, and the obtained results were illustrated with the box plot by Graphpad Prism 8 (https://www.graphpad.com/scientific-software/prism/, accessed on 15 April 2021). The derived graphs were further edited by Adobe Illustrator software CC 2019.

### 4.5. Cis-Element Analyses of GmTALE Gene Promoter Regions

The upstream 2000 bp sequences of the identified *GmTALE* genes were extracted by TBtools [52]. Then the extracted sequences were submitted to PlantCARE (http://bioinformatics.psb.ugent.be/webtools/plantcare/html/, accessed on 15 April 2021) to analyze the *cis*-elements in the gene promoter regions [55]. The diagram of *cis*-elements in the *GmTALE* gene promoter region was depicted by TBtools and was further modified by Adobe Illustrator CC 2019.

### 4.6. Expression Profiling Analyses of GmTALE Genes

The expression profiles of *GmTALE* genes during plant development were obtained from the recorded RNA-seq data on Soybase (https://soybase.org/soyseq/, accessed on 15 April 2021) [38]. The expression profiles of *GmTALE* genes in soybean roots during saline stress and dehydration were derived from published RNA-seq data [39], the RNA-seq reads deposited in Sequence Read Archive database (https://www.ncbi.nlm.nih.gov/sra, accessed on 15 April 2021) under the accession numbers: SRX531069, SRX531070, SRX531071, SRX531072, SRX531073, SRX531074, SRX531075, SRX531076, SRX531077, SRX531078, SRX531079, SRX531080, SRX531081, SRX531082, SRX531083, SRX531084, SRX531085, SRX531086, SRX531087, SRX531088 and SRX531089. And all the transcript levels of the identified *GmTALE* genes were assessed by the RPKM (reads per kilobase per million) values. To better illustrate the expression variations of the identified *GmTALE* genes, the extracted RPKM values were Log_2_ normalized to depict relevant heatmaps of *GmTALE* gene expression profiles by TBtools [52]. To conduct the gene expression correlation analyses, the obtained RPKM values were further submitted to the Omicshare online software (https://www.omicshare.com/tools/Home/Soft/getsoft, accessed on 15 April 2021) to gain the expression correlation matrixes of the *GmTALE* genes. Based on the obtained correlation matrixes, we illustrated associated gene expression correlation heatmaps by TBtools. Cubic heatmaps for representative selected *GmTALE* genes during abiotic stress treatments throughout leaf, stem and root tissues were depicted by TBtools. The output graphs were modified by Adobe Illustrator CC 2019.

### 4.7. Plant Material, Abiotic Stress Treatments and Samplings

Soybean cultivar Williams 82 was adopted as the plant material in this study. Plant material was planted and harvested in 2019 at Dangtu Experimental Station, National Center for Soybean Improvement, Nanjing Agricultural University, Dangtu, Anhui, China. The health and plump soybean seeds were germinated in sterilized vermiculite for three days at 26 °C in the dark. Then the uniform soybean seedlings were transferred into the pots with a mixture of humus and vermiculite (1:2, *v*/*v*). The seedlings were cultivated in a greenhouse at 26 °C with photoperiod 16-h light/8-h dark and 60% relative humidity until the V1 growth stage (the first trifoliate growth stage). For the saline treatment, the seedlings were removed from the pots and held with the foam floats in the 100 mM NaCl solution. For the dehydration treatment, the seedlings were removed from the pots and left in the air at room temperature (22 °C) under water-limiting conditions to impose dehydration stress [39,56]. Samplings were focused on the leaf, stem and root tissues (weighed about 0.1 g of the tissues) after 0, 1, 6, 12 and 24 h after the abiotic stress treatments. Samples collected and frozen in liquid nitrogen and then stored at −80 °C. Five seedlings were set as one independent biological replicate per time point and sampled under different treatments. And three independent biological replicates were conducted at each time point. Total RNA was isolated from the frozen tissues.

### 4.8. RNA Isolations and Quantitative RT-PCR Analyses

Three independent biological replicates were applied for RNA isolations and quantitative RT-PCR analyses. The RNAprep pure plant kit (TIANGEN, Beijing, China) was used for total RNA isolations. The extracted RNA was quality tested by electrophoresis and then quantified with a Nanodrop ND-1000 spectrophotometer (Thermo Scientific, Wilmington, DE, USA) [22]. The HiScript II 1st Strand cDNA Synthesis Kit (Vazyme Biotech, Nanjing, China) was adopted for the conversion of total RNA to cDNA library [22]. To further explore the expression patterns of the *GmTALE* genes in different tissues during abiotic treatments, we selected 12 representative *GmTALE* genes from different GmTALE subfamilies or clades for quantitative RT-PCR analyses. The specific quantitative RT-PCR primers were designed by Primer Premier 5 software (http://www.premierbiosoft.com/primerdesign/, accessed on 15 April 2021). The quantitative RT-PCR assays were operated on a BioRad CFX96 real-time system (CFX96 Touch, Bio-Rad, Hercules, CA, USA) with SYBR qPCR Master Mix (Vazyme Biotech, Nanjing, China) [22]. The housekeeping *GmActin* gene was determined as an internal control. Triplicate quantitative assays were performed on each cDNA sample and analyzed by a 2^−∆∆CT^ method [57].

### 4.9. Statistical Analyses

Student’s *t*-test was performed by Graphpad Prism 8 software. *p*-value cut-off of 0.05 was the criterion to determine whether the test was significantly different or not. All the error bars were standard deviation (SD) from the independent biological replicates.

## 5. Conclusions

In the present study, we identified 68 TALE superfamily members in soybean, which were unevenly distributed on 20 chromosomes and two scaffolds of the soybean genome. The GmTALE members were further evenly divided into the BEL1-like subfamily and the KNOX subfamily. Besides, the KNOX subfamily was separated into Class I, Class II and Class III clades. Gene structures, conserved domain patterns and motif compositions of the GmTALE members in the same subfamily or clade displayed universal similarities, which may indicate their analogous biological functions. Gene duplication analyses demonstrated that segmental duplications took a major role in the expansion of GmTALE superfamily and generating novel *GmTALE* genes. Moreover, the syntenic and evolutionary analyses of the TALE proteins among soybean and multiple species provided more detailed evidence for *GmTALE* gene evolution. *Cis*-element analyses in gene promoter regions, as well as transcriptome data and quantitative RT-PCR investigations, manifested that the *GmTALE* genes play potential roles during soybean development and abiotic stress responses. To conclude, our work laid a foundation for the functional study of *GmTALE* genes in the future, which may enlighten soybean genetic improvement in resistance to saline and dehydration stresses.

## Data Availability

Data is contained within the article or Supplementary Material.

## Supplementary Materials

The following are available online at https://www.mdpi.com/article/10.3390/ijms22084117/s1.

## Abbreviations

aa	amino acid
At	*Arabidopsis thaliana*
BEL1-like	BLH/BELL homeodomain
CDS	Coding sequences
cm	Centimeter
DAF	Days after flowering
Gm	*Glycine max*
Gs	*Glycine soja*
Ka	Non-synonymous substitution
KNOX	KNOTTED-like homeodomain
Ks	Synonymous substitution
ML	Maximum likelihood
MW	Molecular weight
ORF	Open reading frame
Os	*Oryza sativa*
pI	Isoelectric point
RPKM	Reads per kilobase per million
SAM	Shoot apical meristem
Sb	*Sorghum bicolor*
SCW	Secondary cell wall
SD	Standard deviation
Sl	*Solanum lycopersicum*
TALE	Three-amino-acid-loop-extension
UTR	Untranslated region
Vu	*Vigna unguiculata*
