# Supplementary material for: Genome-Wide Identification and Characterization of TALE Superfamily Genes in Soybean (Glycine max L.)
Source: Int J Mol Sci. 2021 Apr 16;22(8):4117. doi: 10.3390/ijms22084117 (PMC8073939; doi:10.3390/ijms22084117)
Supplement: Supplementary file 1 [file ijms-22-04117-s001.zip › Supplementary files/Supplementary Figures.pdf]

Supplementary Figures

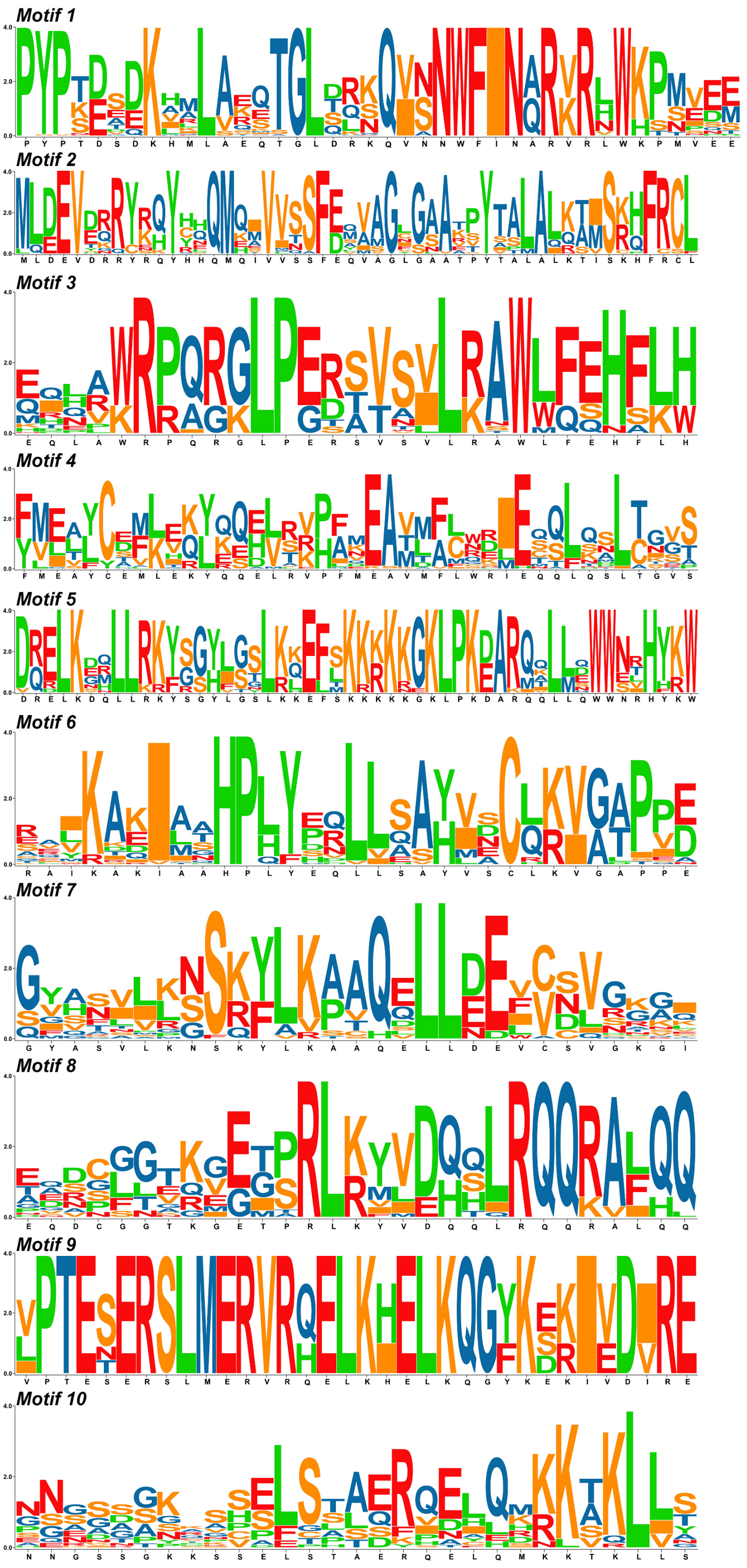

Figure S1. Seq Logos of ten MEME-motifs for the identified GmTALE proteins.

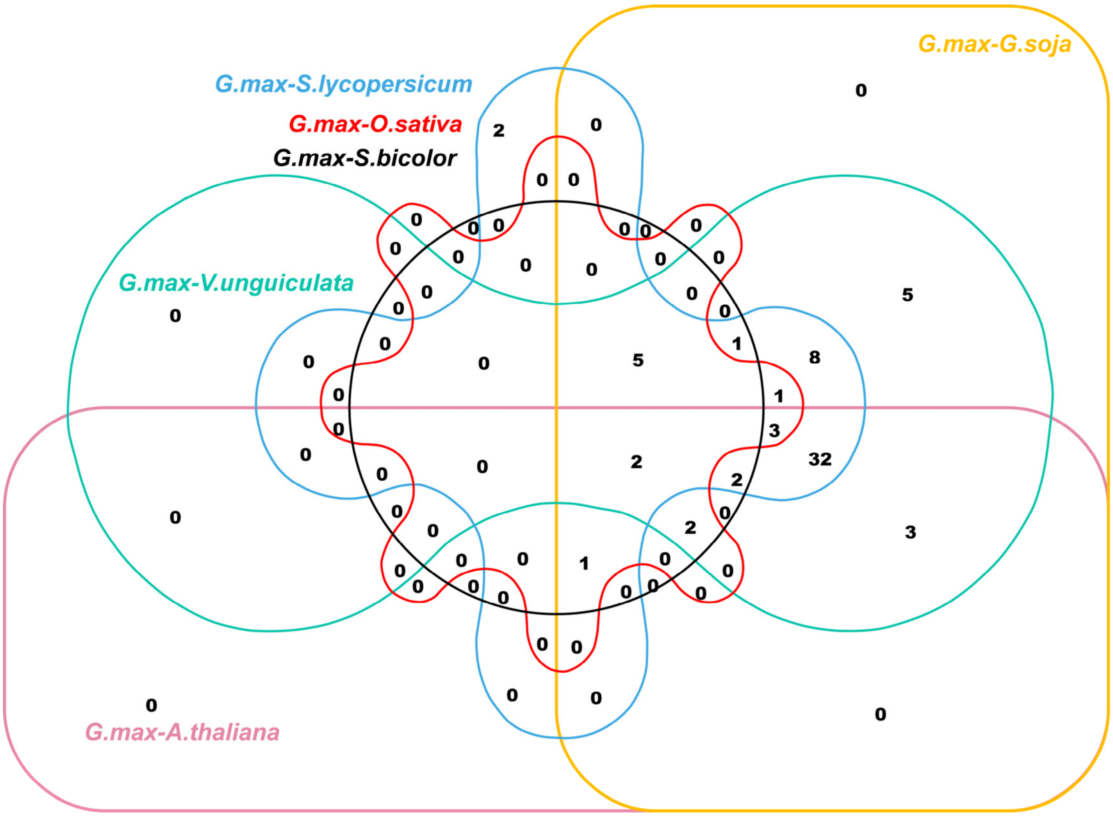

Figure S2. Venn diagram representing non-redundant syntenic TALE members in soybean and other species.

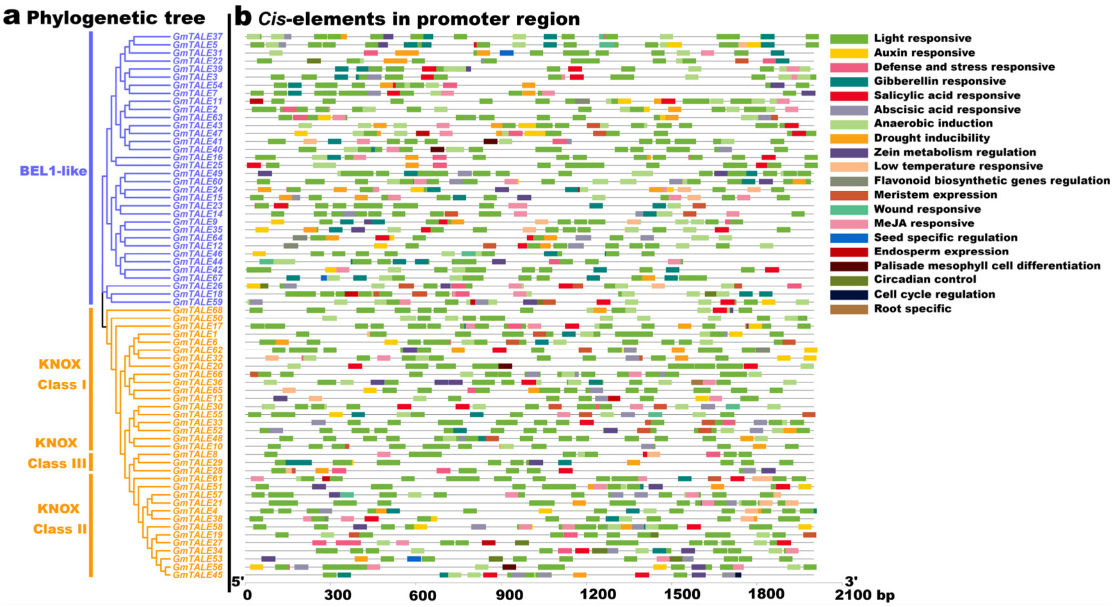

Figure S3. Schematic representing the *cis*-element patterns in the upstream 2000-bp genetic block of *GmTALE* genes. The phylogenetic tree was as built used the maximum likelihood (ML) method with the best scoring JTT + G model.

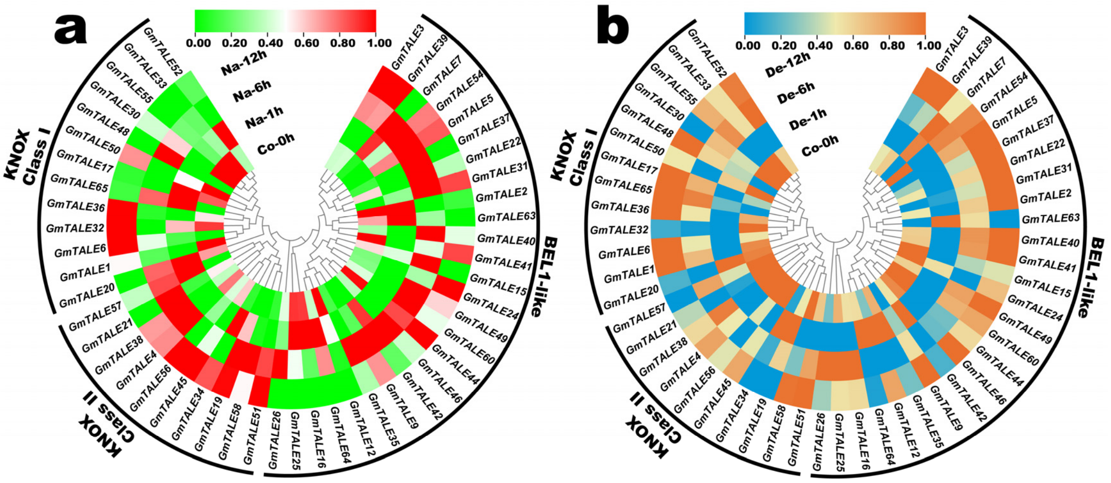

Figure S4. Expression patterns of *GmTALE* genes in the soybean root during saline stress and dehydration.

(a) Hierarchically clustered expression patterns of *GmTALE* genes in the soybean root during saline stress. Na: saline stress; Co: control. **(b)** Hierarchically clustered expression patterns of *GmTALE* genes in the soybean root during dehydration. De: dehydration. The expression data was row-scaled with the zero-to-one method to show the expression pattern of each *GmTALE* gene. The phylogenetic tree was as built used the maximum likelihood (ML) method with the best scoring JTT + G + I model.
